# Phage display as a tool for identifying HIV-1 broadly neutralizing antibodies

**DOI:** 10.18699/VJ21.063

**Published:** 2021-09

**Authors:** A.N. Chikaev, A.N. Chikaev, A.P. Rudometov, Yu.A. Merkulyeva, L.I. Karpenko

**Affiliations:** Institute of Molecular and Cellular Biology of the Siberian Branch of the Russian Academy of Sciences, Novosibirsk, Russia; State Research Center of Virology and Biotechnology “Vector”, Rospotrebnadzor, Koltsovo, Novosibirsk region, Russia; State Research Center of Virology and Biotechnology “Vector”, Rospotrebnadzor, Koltsovo, Novosibirsk region, Russia; State Research Center of Virology and Biotechnology “Vector”, Rospotrebnadzor, Koltsovo, Novosibirsk region, Russia

**Keywords:** phage display, antibody libraries, HIV-1, broadly neutralizing antibodies (bnAbs)., фаговый дисплей, библиотеки антител, ВИЧ-1, нейтрализующие антитела широкого спектра действия (bnAbs)

## Abstract

Combinatorial biology methods offer a good solution for targeting interactions of specif ic molecules by a high-throughput screening and are widely used for drug development, diagnostics, identif ication of novel monoclonal antibodies, search for linear peptide mimetics of discontinuous epitopes for the development of immunogens or vaccine components. Among all currently available techniques, phage display remains one of the most popular approaches. Despite being a fairly old method, phage display is still widely used for studying protein-protein, peptide-protein and DNA-protein interactions due to its relative simplicity and versatility. Phage display allows highly representative libraries of peptides, proteins or their fragments to be created. Each phage particle in a library displays peptides or proteins fused to its coat protein and simultaneously carries the DNA sequence encoding the displayed peptide/protein in its genome. The biopanning procedure allows isolation of specif ic clones for almost any target, and due to the physical link between the genotype and the phenotype of recombinant phage particles it is possible to determine the structure of selected molecules. Phage display technology continues to play an important role in HIV research. A major obstacle to the development of an effective HIV vaccine is an extensive genetic and antigenic variability of the virus. According to recent data, in order to provide protection against HIV infection, the so-called broadly neutralizing antibodies that are cross-reactive against multiple viral strains of HIV must be induced, which makes the identif ication of such antibodies a key area of HIV vaccinology. In this review, we discuss the use of phage display as a tool for identif ication of HIV-specif ic antibodies with broad neutralizing activity. We provide an outline of phage display technology, brief ly describe the design of antibody phage libraries and the affinity selection procedure, and discuss the biology of HIV-1-specif ic broadly neutralizing antibodies. Finally, we summarize the studies aimed at identif ication of broadly neutralizing antibodies using various types of phage libraries.

## Abstract (ru)

Комбинаторная белковая инженерия – востребованный инструмент для решения задач, связанных со скринингом большого разнообразия взаимодействующих молекул: разработки лекарств, средств диагностики, идентификации антител, поиска конформационных имитаторов антигенных детер- минант для создания иммуногенов или компонентов вакцин. Среди всех подобных методик одна из наи- более популярных – технология фагового дисплея, появившаяся во второй половине 1980-х гг., однако в силу относительной простоты и универсальности по-прежнему активно применяющаяся для изучения бе- лок-белковых, пептид-белковых и ДНК-белковых взаимодействий. Фаговый дисплей позволяет создавать высокопредставительные библиотеки пептидов, белков или их фрагментов, в которых каждая фаговая частица экспонирует на своей поверхности исследуемые пептиды или белки и одновременно несет в сво- ем геноме последовательность ДНК, кодирующую экспонируемый пептид/белок. Процедура аффинной селекции позволяет находить специфические фаговые клоны практически к любой мишени, а за счет нали- чия физической связи между генотипом и фенотипом можно эффективно определить структуру отобранных молекул. Значительную роль технология фагового дисплея сыграла в исследованиях, направленных на изучение антигенной структуры вируса иммунодефицита человека (ВИЧ-1) и разработку средств борь- бы с этим заболеванием. Серьезная проблема, из-за которой до сих пор не удается создать эффективную анти-ВИЧ-вакцину, – сильная антигенная изменчивость вируса. Согласно современным представлениям, для обеспечения защиты от инфицирования необходимо стимулировать индукцию в организме вируснейтрализующих антител, активных в отношении большого числа различных штаммов ВИЧ-1. Соответ- ственно, идентификация подобных антител является важной исследовательской задачей. Тема настоящего обзора – применение фагового дисплея в качестве инструмента для поиска ВИЧ-1-нейтрализующих анти- тел широкого спектра действия. Представлены ключевые характеристики технологии фагового дисплея, кратко описан процесс получения библиотек антител, проведения процедуры аффинной селекции, а так- же обсуждается феномен ВИЧ-1-нейтрализующих антител широкого спектра действия. Приводится обзор исследований, посвященных поиску кросс-нейтрализующих антител с использованием различных типов фаговых библиотек.

## Introduction

Phage display was first described in 1985 by George Smith and Gregory Winter, who were awarded the 2018 Nobel Prize in Chemistry for this discovery. They reported that foreign peptides could be successfully expressed on the surface of bacteriophage particles by integrating a gene of interest into a phage genome upstream of its coat protein open reading frame (Smith, 1985). It is noteworthy that a conceptually similar study was independently conducted by a Russian scientific group led by A.A. Ilyichev, who incorporated a peptide-coding sequence into the pVIII protein gene of M13 phage (Ilyichev et al., 1992; Minenkova et al., 1993). Later, G. Smith and colleagues proposed a selection strategy for the enrichment of population of recombinant phage clones that specifically bind to the target ligand, using affinity enrichment process (Smith, 1985). Since there is a direct physical link between the genotype of the recombinant phage particle and the phenotype of the fusion protein, this method allows the identification of DNA sequences encoding selected molecules.

Subsequently, G. Smith and colleagues described the creation of combinatorial phage libraries that contain a large number of phage particles, each carrying a unique protein or peptide on its surface. Currently, one the most common types of phage libraries used for studying various proteinto- protein, receptor-ligand interactions or protein engineering are antibody phage libraries displaying single-stranded (scFv) and antigen-binding (Fab) fragments of IgG molecules (McCafferty et al., 1990; Winter et al., 1994). There are also alternative antibody formats used for the construction of antibody phage libraries, such as variable domains of antibodies from the heavy chains of camelids (VHH, or nanobodies) and sharks (vNAR) (Davies, Riechmann, 1995; Greenberg et al., 1995).

In order to create phage antibody library, antibody fragments to be exposed are usually fused to the N-terminus of the pIII phage coat protein. Despite the fact that all the phage coat proteins can be used for phage display, only pIII is suited to expose large peptides or proteins without loss of infectivity and functional activity of phage particles (Kay et al., 1993; Kishchenko et al., 1994; Mullen et al., 2006; Tikunova, Morozova, 2009). In early phage display systems,gene sequences encoding for antibody fragments were inserted directly into phage genome (McСafferty et al., 1990; Scott, Smith, 1990). Currently, a separate plasmid vector, also known as phagemid, is commonly used to introduce target DNA inserts into the phage genome. Phagemid carries recombinant pIII fusion gene, as well as phage and bacterial replication origins (thus can be replicated independently of phage production), but it lacks phage genes necessary for infecting, replicating, assembling and budding phage particles. In order to produce recombinant phages, phagemidtransformed Escherichia coli cells should be coinfected with a helper phage that carries wild-type phage genome including all the remaining phage genes required for the phage life cycle (Ledsgaard et al., 2018). The phage origin of replication in the phagemid enables its packaging into the forming virions as a single-strand DNA. Thus, the resulting phage particles contain both recombinant and wild-type forms of pIII from the helper-phage, so the infectivity is not compromised (Felici et al., 1991). 

Immune libraries are usually generated from B-cell derived antibody repertoire of immunized animals or reconvalescent donors. Phage immune libraries contain about 107–108 unique phage clones displaying antigen-specific antibodies on their surface (Kennedy et al., 2018). In some cases, “naive” libraries, based on lymphocyte mRNA of unvaccinated/ healthy donors, or intact animals, as well as “synthetic” libraries, based on de novo synthesized oligonucleotides, may be used in order to enhance diversity of the antibody repertoire (Griffiths, Duncan, 1998; Tikunova, Morozova, 2009). The representativeness of the phage libraries can reach up to 109–1010 for “naive” and 1010–1011 for “synthetic” libraries (Zhao et al., 2016; Kennedy et al., 2018; Muyldermans, 2021).

Construction of antibody phage display libraries

The library’s construction begins with RNA isolation from hybridoma cell lines, spleen cells from immunized animals, or B-lymphocytes from human peripheral blood, and subsequent cDNA synthesis (Clackson et al., 1991). Then, using isotype-specific primers, the variable regions of immunoglobulin light (VL) and heavy (HV) chain genes (or solely VH in the case of VHH) are amplified and cloned into a phagemid vector between the pIII-encoding gene and N-terminal signal sequence, which directs fusion protein to periplasmic translocation. These phagemids encoding diverse VH/VL gene combinations are then used for the transformation of E. coli cells that are co-infected with a helper phage, leading to the production of a set of phage particles exposing different antibody fragments (Skerra, Pluckthun, 1988; Tikunova, Morozova, 2009; Hammers, Stanley, 2014).

cting phage clones that carry antigen-specific variants of antibody fragments) is carried out. To do this, the library is incubated with the target antigen that is immobilized on an immune plate, magnetic beads, or immunosorbent. Unbound particles are then washed, and a fraction of target-specific phages can be eluted using buffer with low or high pH or by adding a competing protein or peptide that strongly interferes with binding of the target molecule to selected phages (Smith, Petrenko, 1997). Another frequently used method of biopanning, probably the most effective, involves affinity selection using target molecules labeled with SS-biotin (Chames, Baty, 2010). The target molecule must be immobilized via a streptavidin-coated template. After that, the procedure involves standard incubation of the phage library and washing the unbound phage clones. SS-biotin contains a disulfide bond that can be cleaved by treatment with sulfhydryl, which enables the separation of a complex target (specific phage) from the substrate by adding a reducing agent such as dithiothreitol or 2-mercaptoethanol. This method provides a significant increase in the percentage of target-specific clones during biopanning, since the eluate obtained this way doesn’t contain any phages that are nonspecifically bound to the substrate.

After each round of biopanning, eluted phage clones are used to infect E. coli, which are then cultured and superinfected with helper phage. The produced progeny phage particles are used for subsequent rounds of affinity selection. Commonly, one or two rounds of panning are enough to enrich the library with antigen-specific phage clones, though in the case of synthetic libraries (which are more representative but less specific) the number of rounds might be increased up to five.

Phage titering is done after every affinity screening to assess the amount of target-specific clones. The specificity of each phage clone can be assessed using enzyme-linked immunosorbent assay (ELISA), immunoblotting or flow cytometry. After the final round of biopanning, phages with the highest affinity are picked up, amplified, sequenced and used for phage DNA extraction and amplification of VHH, Fab- or scFv-coding sequences, which are then subcloned into an expression vector in order to express the soluble forms of corresponding proteins. Such Fab/scFvs can also be converted into full-length monoclonal antibodies (mAbs) by in-frame cloning of VH and VL genes into the cassette vector that harbors appropriate heavy IgG constant region genes.

**Fig. 1.**
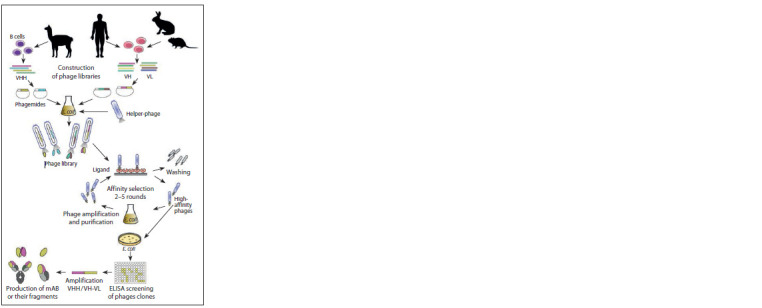
Scheme representing generation of phage display antibody libraries and biopanning procedure.

In the final step, identified antibodies should be validated for their avidity and affinity against the target antigen via ELISA, western blotting or another immunological assay (Alfaleh et al., 2020). The general scheme of the method is shown in Fig. 1.

The number of panning rounds can vary depending on whether a greater variety of clones or a greater specificity of fusing to an antigen is required. Additionally, double recognition panning against two antigens can be carried out for the selection of bispecific antibodies (Hammers, Stanley, 2014).

Phage display has plenty of applications: it is used for the development of antibacterial therapeutic agents (Christensen et al., 2001; Huang et al., 2012; Ashby et al., 2017), biosensors (Moon et al., 2019; Sozhamannan, Hofmann, 2020), identification of mAbs for treatment of dermatological, autoimmune diseases or cancers (Chan et al., 2014; Hammers, Stanley, 2014; Nixon et al., 2014; Alfaleh et al., 2020), as a platform for targeted drug and vaccine delivery (Clark, March, 2004; Petrenko, Jayanna, 2014; Nemudraya et al., 2016), as a tool for diagnostics and treatment of viral infections (Castel et al., 2011; Hess, Jewell, 2020). Phage display also has broad applications in the field of HIV-1 research: mapping epitopes recognized by HIV-neutralizing antibodies; searching for HIV-derived peptide mimics, which could be used a fusion inhibitors, components of vaccines and diagnostics; identifying HIV-neutralizing antibodies with broad neutralizing activity.

Below we review some examples of identification of HIV-1 broadly neutralizing antibodies using phage display technology.

Broadly neutralizing antibodies

One of the prominent features of human immunodeficiency virus is its phenomenal ability to evade the humoral immune response by rapidly mutating due to the low fidelity of HIV-1 reverse transcriptase which markedly enhances the genetic variation of the virus and makes it mutate very quickly – at the highest rate for any biological entity (Cuevas et al., 2015). Mutations occurring after each cycle of viral replication often cause structural changes in HIV immunodominant regions. As a result, the majority of antibodies elicited against HIV infection are strain-specific, and either are non-neutralizing or lose the ability to neutralize the virus after several replication cycles due to antigen escape. In this regard, it was believed that HIV-1 neutralizing antibodies could not be induced or it occurs extremely rarely (Mccoy, Burton, 2017). Nevertheless, these antibodies were lately found in so-called HIV long-term non-progressors – HIV-infected patients who do not develop immunodeficiency in the absence of antiretroviral therapy. Sera of non-progressors exhibited HIV-neutralizing activity not only against host strains, but also against a panel of different HIV-1 isolates (Dhillon et al., 2007; Walker et al., 2010; Sok, Burton, 2018; Dashti et al., 2019).

It was originally thought that induction of antibodies with broad HIV-1 neutralizing activity is a unique feature of nonprogressors which provide them with the ability to control viremia for a long time (Montefiori et al., 1996). Later, it was shown that bnAbs is elicited in 20–50 % of all HIV-1 infected patients, but it takes a very long time before mature neutralizing antibodies can be arisen: the affinity maturation process may last up to several years from the moment of infection (Doria-Rose et al., 2009; Hraber et al., 2014; Rusert et al., 2016). It was shown that passive administration of a single bnAbs or its combinations to non-human primates completely protected animals against SHIV infection (Hessell et al., 2009; Moldt et al., 2012; Shingai et al., 2014). Moreover, passive transfer of bnAbs to HIV-infected individuals correlated with a long-term viral load reduction to undetectable levels (Lynch et al., 2015; Scheid et al., 2016), and in some cases, a host-protective humoral immune response was formed (Schoofs et al., 2016).

Today, the majority of HIV researchers acknowledge that an immunogen capable of eliciting broadly neutralizing antibodies may provide protection against HIV. Thus, the search for bnAbs and the development of immunogens aimed at elicitation of broadly neutralizing antibodies are among the most important tasks of modern vaccinology.

Phage display as a tool for identification of HIV-1 broadly neutralizing antibodies

**Fig. 2.**
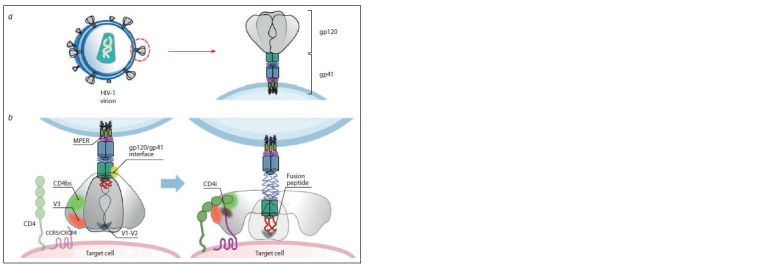
Sites of HIV-1 vulnerability to bnAbs. a – schematic representation of HIV-1 envelope glycoprotein (Env). Left – HIV-1 virion structure, right – mature HIV-1 envelope (Env) protein anchored to the viral membrane. Env is a trimer of heterodimers composed of gp41 (transmembrane subunit) and gp120 (surface subunit) molecules; b – conformational changes of HIV-1 Env trimers occurring during infection of host cell. Known HIV-1 sites of vulnerabilities to broadly neutralizing antibodies are indicated: gp120 binding site (CD4bs); V1/V2 glycan apex, loops V3 glycan loop of gp120 (V1/V2, V3); CD4-induced CCR5/CXCR4 binding site which is exposed following CD4 binding (CD4i); gp120/gp41 interface; membraneproximal external region of gp41 (MPER); gp41 N-terminal fusion peptide, which anchors to the host cell membrane.

The first studies devoted to the identification of bnAbs using phage display were published in the early 1990s. At that time, there were practically no data on broadly neutralizing antibodies. However, detailed information about the antigenic structure of HIV-1 had already been obtained, which resulted in the understanding that in order to provide effective protection against the virus, the humoral immune response must be targeted to the conserved viral epitopes, which are less susceptible to mutagenesis (Kowalski et al., 1987; Habeshaw et al., 1990; Putney, 1992). These fragments have been considered as the main targets for neutralizing antibodies. Subsequently, other HIV-1 antigenic determinants were discovered, which are critical for the HIV entry into the host cells, also known as sites of vulnerability (Shcherbakov et al., 2015; Kwong, Mascola, 2018). Up to date, at least seven sites on HIV Env that are vulnerable to antibody-mediated protection have been identified (Fig. 2).

bnAbs recognizing conserved regions of the gp120 glycoprotein

IgG1b12 was the first HIV-1 broadly neutralizing antibody derived using phage display (and also one of the first described bnAbs). In 1991, D.R. Burton et al. obtained an immune Fab library of bacteriophages based on B-cells from the bone marrow of an HIV-positive non-progressor (Barbas et al., 1991; Burton et al., 1991). After the panning of the resulting library against the HIV-1 IIIB gp120 glycoprotein, they selected phage clones that specifically bound with gp120. As a result, specific combinations of VH: VL genes were identified and expressed in the Fab format. It was shown that these Fabs were able to compete with the soluble CD4 molecule (sCD4) for binding to gp120 in ELISA (Burton et al., 1991). In their next work, the authors demonstrated the ability of selected Fabs to neutralize IIIB, MN and RF HIV-1 strains (Barbas et al., 1992).

Expanded screening of the phage library and more detailed analysis revealed a clone displaying the Fab fragment numbered b12, which binds with high affinity to the mature form of gp120 at the CD4bs (Roben et al., 1994). Later, a full-length recombinant IgG1b12 antibody was obtained, which became one of the first HIV-1 broadly neutralizing antibodies discovered (Burton et al., 1994).

Neutralization breadth of IgG1b12 has been repeatedly evaluated using different viral strains and panels of primary HIV-1 isolates. Depending on the panel used, b12 neutralized 30–63 % of the pseudovirus/primary isolates panels used in the experiment (at a concentration of IC50 < 50 μg/ml), whereas the highest neutralization potency was demonstrated against HIV-1 subtype B (clade B HIV-1) (Burton et al., 1994; Walker et al., 2009; Corti et al., 2010; Wu et al., 2010; Zhang et al., 2012; Gach et al., 2013). Before the advent of the second generation bnAbs, obtained by sorting affinity B-cells memory (Sok, Burton, 2018), among all detected at that time cross-neutralizing antibodies IgG1b12 was one of the leaders in the number of neutralized isolates HIV-1.

bNAbs specific to CD4 binding site

M.Y. Zhang et al. were the first researchers who identified two CD4bs-specific broadly neutralizing antibodies. They constructed Fab phage immune libraries derived from the bone marrow B-cells of an HIV-infected non-progressor who had high titers of HIV-1-specific broadly neutralizing antibodies

In order to identify clones that bind to the conserved antigenic determinants of the virus, biopanning was performed using two antigens. The first round of selection was carried out against sCD4 in complex with recombinant gp14089.6 – a truncation form of gp160 Env of HIV-1 89.6 strain with removed transmembrane and cytoplasmic domains. In the second round, library was panned against HIV-1 IIIB gp140 and sCD4 complex. Subsequent selection rounds were carried out against gp14089.6 and gp140IIIB molecules, respectively, with a gradual decrease in antigen concentration at each round. The binding affinity of the selected clones was evaluated by ELISA using single gp14089.6/gp140IIIB molecules or in complex with sCD4.

The selected m18 clone showed the highest binding affinity to all of the antigens and was capable of neutralizing 11 out of 15 pseudoviruses bearing HIV-1 Envs from different strains (Zhang et al., 2003). A year later, the selected clones were re-screened by ELISA using the additional JR-FLgp120 antigen, resulting in selection of m14 Fab clone with enhanced affinity and neutralization breadth compared to m18 (Zhang et al., 2004a). Next, these clones were tested for neutralization activity against extended panel of 30 HIV isolates. It was shown that m14 and m18 were able to neutralize about 21–23 % and 13–21 % of a panel, respectively, thereby demonstrating lower neutralization breadth compared to bnAb IgG1b12 (Zhang et al., 2012).

Using a similar approach, the same research group screened a Fab phage library derived from B-cells of R2 donor with high titers of cross-neutralizing antibodies. Two of the identified clones, m22 and m24, were expressed as Fab fragments of CD4bs-specific antibodies, which demonstrated neutralization potency and breadth similar to m14 and m18 (Zhang et al., 2006).

bNAbs specific to CCR5/CXCR4 coreceptor-binding sites of gp120

M. Moulard et al. screened a Fab antibody phage library (IgG1κ) derived from an HIV-infected individual against the gp120-CD4-CCR5 complex (Moulard et al., 2002). After five rounds of affinity selection, a Fab clone X5 with a unique CDR3 heavy-chain was selected. It was shown that the binding affinity of X5 to the CD4-gp120/CD4-gp140 complexes was significantly higher than that to the single gp120 and gp140 molecules, respectively. Addition of denatured CCR5 to the CD4-gp120 complex increased the X5 affinity, indicating that its epitope is formed by a CD4-dependent conformational change of gp120. X5 partially competed for binding to gp120 with other CD4i-specific antibodies, and with CD4bs-recognizing bnAb IgG1b12. Furthermore, X5 Fab neutralized 11 out of 12 primary HIV-1 isolates, thus demonstrating affinity, breadth, and potency comparable to the full-length IgG1b12 (Moulard et al., 2002).

However, the hypothesis that the full-length bivalent variant of IgG X5 would have even greater neutralizing activity was not confirmed. Apparently, the availability of the epitope recognized by X5 is limited by steric hindrance, which may cause the lack of binding efficacy of the larger molecules (Labrijn et al., 2003; Choudhry et al., 2006). It was thus concluded that the single-chain fragment of this X5 antibody possesses the highest activity and neutralization breadth, compared to its Fab and IgG variants (Choudhry et al., 2006).

Later, non-specific mutagenesis of the X5 scFv-encoding sequences was carried out, the resulting “mutant” phage sublibrary was screened against the oligomeric form of gp14089.6 (which was non-homologous to gp120JR-FL) in complex with sCD4. Two scFvs, m6 and m9, capable of neutralizing 96 and 100 % of primary isolates from a panel comprising 33 different strains, were identified. Moreover, X5 neutralized only 45 % of the isolates from this panel (Zhang et al., 2004b). Neutralization assay performed using another panel of 30 HIV-1 different strains revealed that m9 neutralized 76 % of the primary isolates (Zhang et al., 2012).

bNabs specific to the MPER region of the gp41 glycoprotein

Another site of vulnerability of HIV-1, the membrane-proximal outer region of the gp41 glycoprotein (MPER), which is located between the transmembrane region and the gp41 C-terminal α-helical fragment, became another target of HIV bnAbs. Since MPER plays a crucial role in the process of viral fusion to the target cell, it is highly conservative and thus considered as one of the most promising targets for the development of antiviral drugs (Burton, Hangartner, 2016). The study performed by M. Zwick and his colleagues (2001) should be mentioned as one of the first attempts to search for MPER-specific bnAbs using phage display. They developed an immune Fab phage library based on cDNA of VH/VL genes isolated from the B cells of the bone marrow of an HIV-1 non-progressor who had a high titer of broadly neutralizing antibodies.

Two biopanning strategies were applied: in the first case, the HIV-1MN gp41 peptide MN 2031 comprising the MPER sequence was used as an antigen. In the second, the selection was carried out against a whole HIV-1MN virion. After the panning, several MPER-specific clones were identified, including Fab Z13 clone which had the highest affinity and neutralization breadth. Next, the authors created a “mutant” phage library displaying Fab Z13 with random mutations in the LCDR3 and screened it against the gp41 glycoprotein, in order to identify the Z13 clones with enhanced affinity. Among the selected phages, clone exposing Fab Z13e1 bound to gp41 MPER with the highest affinity. Subsequently, a full-length Z13e1 IgG molecule was obtained, which provided a more-than-100-fold enhanced affinity for binding to MPER, and a significant increase in HIV-1-neutralizing activity compared to the initial IgG Z13 variant. The amount of neutralized isolates increased from 35 to 50 % (Zwick et al., 2001; Nelson et al., 2007).

bNabs recognizing the gp120–gp41 interface

Phage display was also used for identification of bnAbs that bind to the N-terminal domain of gp41, the so-called fusion peptide (see Fig. 2). Antibodies are able to bind to gp41 after conformational changes occurring during the last stages of HIV cell entry.

Such anti-gp41 antibodies were first described by M. Miller’s research group (Miller et al., 2005). They used B-cells from the bone marrow of an HIV-negative patient for naive scFv phage library construction and subsequent identification of human monoclonal antibodies specific to the gp41 N-terminal region (NHR). The library was subsequently panned against the polymer that mimics gp41 6HB peptide complex, and then against the IZN36 peptide mimetic of the N-terminal heptad repeats (NHR). As a result, both antigen- binding phage clones were selected and used for reconstruction of corresponding soluble scFvs and full-length IgGs. Virus neutralization assay led to the detection of H/I1-BMV-D5 antibody capable of neutralizing 9 of 19 tested HIV-1 isolates (Miller et al., 2005).

Searching for bnAbs using phage display of single domain antibody fragments

In addition to the “classical” scFv/Fab phage display, phage libraries based on camelid single domain antibodies (nanobodies) were used to search for HIV bnAbs (see Fig. 1). Compared to IgG, nanobodies are more stable and have a smaller size, which can be beneficial for binding to sterically restricted antigenic determinants. Besides, the absence of light chains in the VHH structure facilitates gene manipulations and cloning procedures during library construction.

One of the first immune VHH phage libraries was obtained from llamas immunized with trimeric form of HIV-1 gp140 derived from a clade C (CN54). Total RNA of lymphocytes was isolated and used for cDNA synthesis; the repertoire of VHH genes was amplified and cloned into a phagemid vector, obtaining a library of phages displaying HIV-specific VHHs as pIII fusion proteins. Biopanning of the library led to the isolation of VHH A12, C8 and D7 that were able to neutralize 24 and 26 of 65 HIV-1 Env-pseudotyped virions from tier 1, 2, and 3 of various isolates (Forsman et al., 2008). Similarly, a phagemid immune library based on a VHH from llama immunized with clades A and B/C HIV-1 gp140 was obtained. After the biopanning, J3 and 3E3 clones were identified, which bound specifically to CD4bs and were able to neutralize 96 and 95 % of the pseudovirus panel (Mccoy et al., 2012, 2014; Strokappe et al., 2012).

Later, the same library was used for selection of VHH clones that were specific to the other HIV-1 glycoproteins:1F10, which binds to the V3 loop of gp120; 1B5, which recognizes the CCR5 binding region, gp41-specific 2H10 and 2E7 clones. These nanobodies were capable of neutralizing from 45 to 80 % of Env pseudoviruses from the panels used (Lutje Hulsik et al., 2013; Strokappe et al., 2019). Bivalent nanobodies carrying VHHs with the highest neutralizing activity were also designed. The neutralization potency of these bispecific nanobodies increased approximately 1400- fold compared to the mixture of the individual VHHs; the highest efficiency of the nanobodies was observed against clade C HIV-1 viral strains (Lutje Hulsik et al., 2013; Strokappe et al., 2019). 

K. Koch and colleagues (2017) separately prepared a phage immune library of HIV-specific VHHs using lymphocytes of camel immunized with soluble stabilized HIV-1 clade C gp140 Env trimer (SOSIP gp140). After affinity selection of the library, several CD4bs-specific nanobodies were identified, the best of which (VHH-9, VHH-28, VHH-A6) were capable of neutralizing 53, 65, and 77 % of a 21-isolate HIV-1 Env pseudovirus panel.

The authors of the above-mentioned studies emphasize that display of nanobody immune libraries via phage display is a convenient and effective alternative to the “traditional” scFv/Fab libraries for searching for high-affinity HIV-1 broadly-neutralizing antibodies. Small size and chemical stability of VHH facilitate various genetic manipulations directed to obtain clones with improved characteristics, such as site-targeted mutagenesis or creation of humanized and multivalent nanobodies specific to different regions of viral antigens. Finally, VHH phage libraries may be considered as a cheaper-to-manufacture alternative to full-sized human MAbs for HIV treatment (Weiss, Verrips, 2019).

## Conclusion

Phage display technology played an essential role as a tool for searching, studying and epitope mapping of HIV-neutralizing antibodies. Phage display yielded the first HIV-1 bnAb, thereby leading to the extensive development of this research area. Soon, broadly neutralizing antibodies became a major focus of HIV vaccine design. Current methods for isolating HIV-specific bnAbs include the sorting of antigen-specific B cells with one memory on virus-like particles or variable loop removed recombinant viral proteins (Wu et al., 2010). These techniques, along with high-throughput screening of selected antibody clones (Walker et al., 2009), allowed the identification of second-generation HIV-1 bnAbs with markedly increased potency and breadth. The discovery of such antibodies capable of neutralizing more than 90 % of viral isolates has reinvigorated interest in the use of bnAbs in HIV-1 therapy

Since 2010, more than 30 clinical trials of broadly neutralizing antibodies have been registered (Mahomed et al., 2021). Among them, 12 studies successfully passed Phase I, demonstrating safety of bnAbs and their combinations; four Phase II bnAb trials are currently underway, first data are expected to be made publicly available in 2021 (Julg, Barouch, 2019; Karuna, Corey, 2020; Mahomed et al., 2020;Stephenson et al., 2020). Lastly, relying on the progress achieved in generating recombinant viral antigens (Jardine et al., 2013; Medina-Ramirez et al., 2017; Stamatatos et al., 2017; Duan et al., 2018), together with a vast amount of data accumulated on broadly neutralizing antibodies (Mascola, Haynes, 2013; Mouquet, Nussenzweig, 2013), novel strategies for design of HIV vaccines aimed for induction of 2nd generation bnAbs have been proposed (Del Moral-Sanchez, Sliepen, 2019). 

Hence, bNAbs represent a promising novel approach for effective HIV-1 immunotherapy and prevention. Thus, today bnAbs are one of the most important objects in the study of HIV infection. It is likely that in the foreseeable future they will become a worthy alternative to existing antiretroviral therapy, and in the longer term, one can expect the emergence of preventive vaccines that induce their production.

## Conflict of interest

The authors declare no conflict of interest.
